# ^99m^Tc-Labeled Diarylpyrazoles for Single-Emission Computer Tomography Imaging of Neurotensin Receptor-Positive Tumors: A Comparative Preclinical Study

**DOI:** 10.3390/pharmaceutics17060700

**Published:** 2025-05-27

**Authors:** Roman Potemkin, Simone Maschauer, Harald Hübner, Torsten Kuwert, Tobias Bäuerle, Peter Gmeiner, Olaf Prante

**Affiliations:** 1 Department of Nuclear Medicine, Molecular Imaging and Radiochemistry, Translational Research Center, Friedrich-Alexander-Universität Erlangen-Nürnberg (FAU), 91054 Erlangen, Germany; roman.potemkin@fau.de (R.P.); simone.maschauer@uk-erlangen.de (S.M.); torsten.kuwert@uk-erlangen.de (T.K.); 2 Bavarian Cancer Research Center (BZKF), Translational Research Group TRAFO, 91054 Erlangen, Germany; 3 Department Chemistry and Pharmacy, Medicinal Chemistry, Friedrich-Alexander-Universität Erlangen-Nürnberg (FAU), 91058 Erlangen, Germany; harald.huebner@fau.de (H.H.); peter.gmeiner@fau.de (P.G.); 4 Department of Radiology, Preclinical Imaging Platform Erlangen (PIPE), Friedrich-Alexander-Universität Erlangen-Nürnberg (FAU), 91054 Erlangen, Germany; 5 Department of Radiology, University Medical Center Mainz, Johannes-Gutenberg University, 55131 Mainz, Germany; tobias.baeuerle@unimedizin-mainz.de; 6 FAU NeW-Research Center New Bioactive Compounds, Friedrich-Alexander-Universität Erlangen-Nürnberg (FAU), 91058 Erlangen, Germany

**Keywords:** neurotensin receptor, SPECT imaging, technetium-99m, molecular imaging, pancreatic cancer

## Abstract

**Background/Objectives:** Neurotensin receptors (NTSRs), members of the G protein-coupled receptor (GPCR) family, have been found to be overexpressed in several types of human cancers, including breast, colon, lung, liver, prostate, and pancreatic cancer. In particular, NTSR1 is overexpressed in at least 75% of pancreatic ductal adenocarcinomas. The aim of the present study was the development and evaluation of new ^99m^Tc-labeled nonpeptide NTSR1-antagonists for SPECT imaging of NTSR-positive tumors. **Methods:** Multistep syntheses of NTSR1 antagonist derivatives were performed following our previously described procedure. Two different chelating strategies were applied for ^99m^Tc radiolabeling to provide the [^99m^Tc]Tc-HYNIC complex **[^99m^Tc]1** and the [^99m^Tc]Tc-tricarbonyl complex **[^99m^Tc]2**. Receptor binding assays were performed using hNTSR1-expressing CHO cells. Radiochemical yields (RCYs) were determined by radio-HPLC. For **[^99m^Tc]1** and **[^99m^Tc]2**, log *D*_7.4_, plasma protein binding, stability in human plasma and serum, and cellular uptake in HT-29 cells were determined. Biodistribution studies and small animal SPECT studies were performed in HT-29 tumor-bearing nude mice. **Results:** The radiosynthesis of **[^99m^Tc]1** (log *D*_7.4_ = −0.27) and **[^99m^Tc]2** (log *D*_7.4_ = 1.00) was successfully performed with RCYs of 94–96% (decay-corrected). Both radioligands were stable in human serum and plasma, showed plasma protein binding of 72% (**[^99m^Tc]1**) and 82% (**[^99m^Tc]2**), and exhibited high and specific uptake in HT-29 cells. Biodistribution studies in HT-29 tumor-bearing mice showed a higher tumor accumulation of **[^99m^Tc]1** compared to **[^99m^Tc]2** (8.8 ± 3.4 %ID/g vs. 2.7 ± 0.2 %ID/g at 2 h p.i.). **[^99m^Tc]2** showed exceptionally high intestinal accumulation (49 ± 22 %ID/g at 1 h p.i.) and was therefore considered unfavorable. In the SPECT/CT imaging of HT-29 tumor xenografts, **[^99m^Tc]1** showed a higher NTSR1-specific tumor uptake than **[^99m^Tc]2** at all time points after tracer injection, with 12 ± 2.8 %ID/g for **[^99m^Tc]1** vs. 3.1 ± 1.1 %ID/g for **[^99m^Tc]2** at 4 h p.i. and adequate tumor-to-background ratios. **Conclusions:** In particular, the [^99m^Tc]Tc-HYNIC ligand (**[^99m^Tc]1**) showed promising preclinical results, being a potential candidate for SPECT imaging and, therefore, appropriate for translation into the clinic.

## 1. Introduction

Neurotensin (NTS) is a 13-amino-acid peptide originally isolated from bovine hypothalamic and characterized by Carraway and Leeman in 1973 [1]. NTS exerts its biological effects mainly through the interaction on cell surfaces with two 7-transmembrane class A G protein-coupled receptors (GPCRs), namely neurotensin receptor 1 (NTSR1) and NTSR2. A third receptor, NTSR3, has been identified, being identical to the previously reported sortilin protein [2]. It was discovered that the C-terminal sequence NTS(8–13) (H-Arg-Arg-Pro-Tyr-Ile-Leu-OH) is the essential NTSR binding sequence. NTS binds with high affinity to NTSR1 (K_i_ = 0.15–0.5 nM) and with lower affinity to NTSR2 (K_i_ = 5–7 nM) [3]. In 2012, White et al. published the first crystal structure of the rat NTSR1 bound to NTS(8–13) [4], providing important insights into the binding mode and supporting the development of NTSR1 ligands. NTSR1 expression has been reported to be overexpressed in several types of human cancers, including breast [5], colon [6], lung, liver, prostate, and pancreatic cancer [7,8,9]. Remarkably, NTSR1 is highly expressed on 75% of pancreatic ductal adenocarcinomas (PDACs) [9,10]. PDAC is the eighth leading cause of cancer death in the world, with the poorest prognosis amongst all human malignant solid tumors due to its fast doubling time and high rate of metastasis, mainly in the liver and peritoneal cavity. Today, the treatment of choice for resectable pancreatic carcinoma is surgery; however, patients suffering from PDAC show a highly disappointing 5-year survival rate of 10%, and their median overall survival ranges from 9 to 10 months. Therefore, the development of new potent diagnostic and therapeutic radioligands for the early detection of pancreatic carcinoma by sensitive imaging modalities, such as positron emission tomography (PET) or single-emission computer tomography (SPECT), and effective endoradiotherapy is highly desirable [11].

Up to now, various radiolabeled (^68^Ga, ^18^F, and ^64^Cu) peptides agonist candidates targeting NTSR1 have been studied [12,13,14,15,16,17]; however, it is commonly known that they possess unfavorable in vivo properties, such as degradation by peptidases, resulting in short half-lives in vivo or high uptake in the kidneys [17,18]. A translation into routine diagnostic human application has not yet been reported.

**Figure 1:**
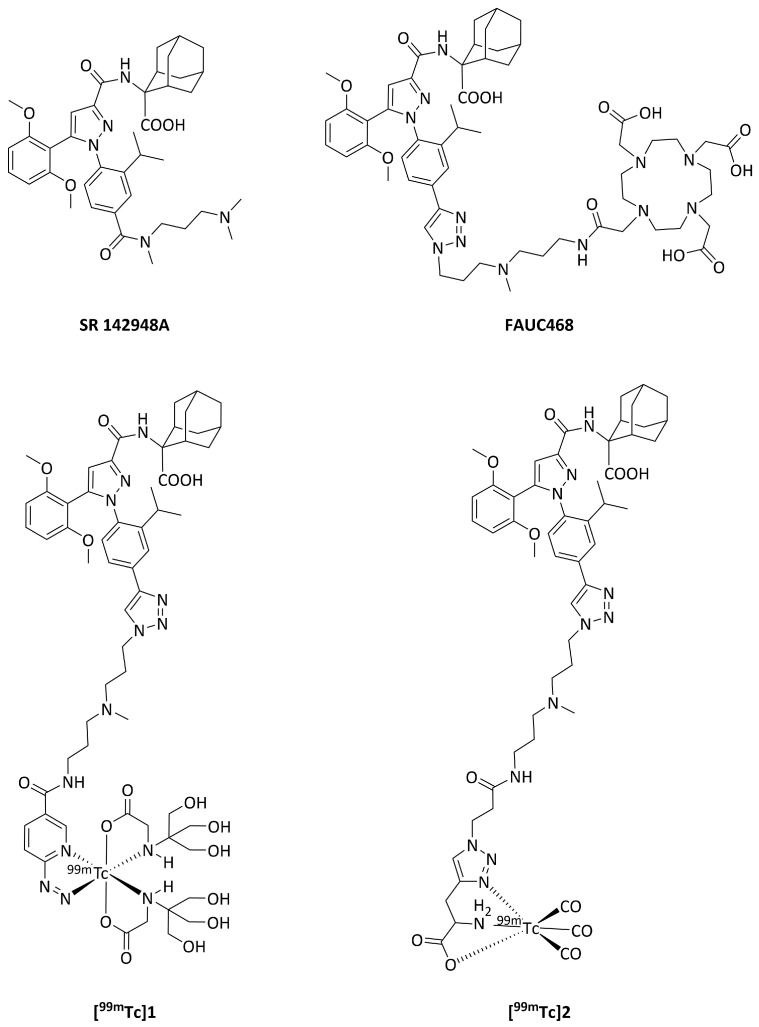
Chemical structures of SR 142948A, FAUC468 [28], **[^99m^Tc]1**, and **[^99m^Tc]2**.

Contrarily, nonpeptide antagonists demonstrated highly improved properties in vitro with their subnanomolar NTSR1 affinity and in vivo with their low uptake in the kidneys [17,19]. Moreover, improved knowledge of their NTSR1 binding mode offers a solid basis for the optimization of ligands [20]. In 1993, Gully et al. introduced SR 48692 (Meclinertant, Sanofi S.A., Paris, France), the first potent and selective nonpeptide antagonist targeting NTSRs [21]. SR 48692was discovered by random screening of several thousand chemicals and exhibited nanomolar NTSR1 affinity in different receptor-positive tissues and cells from various species (e.g., IC_50_ = 30 nM, HT-29 cells, [^125^I-Tyr^3^]NT(8-13) as radioligand) and selectivity over NTSR2 [21,22]. SR 142948A (Figure 1), structurally related to SR 48692 [23], revealed improved solubility in water and higher NTSR affinity than SR 48692 (e.g., IC_50_ = 0.32 nM, HT-29 cells) without NTSR subtype selectivity [19,23]. In our previous work based on the lead SR 142948A, we developed the first ^18^F-fluoroglycosylated NTSR1 antagonist (K_i_ (NTSR1) = 0.98 nM) with excellent biodistribution, rapid clearance from blood, and specific NTSR1-mediated tumor uptake in HT-29 tumor-bearing mice, as demonstrated by PET imaging [24]. The introduction of the metal chelator dodecane tetraacetic acid (DOTA) instead of the glycosyl moiety linked to the diarylpyrazole antagonist, enables radiolabeling with gallium-68, indium-111, or luthetium-177.

However, DOTA derivatives showed significantly increased plasma protein binding, resulting in a slow steadily increasing tumor uptake over time, so that the short half-life of gallium-68 (t_½_ = 68 min) did not appear to be biocompatible due to the low tumor-to-blood ratio at 60 min p.i. [17]. The DOTA derivative 3BP-227 radiolabeled with long-lived indium-111 (t_½_ = 2.8 d) indicated specific HT-29 tumor uptake with adequate tumor-to-blood ratio at 6 h p.i. [25]. Therefore, ^177^Lu-3BP-227 was successfully applied to preclinical therapy studies and first-in-human studies for the radiotherapy of pancreatic carcinoma in six patients [26,27]. In our own previous work, we have developed the triazolyl NTSR1 antagonist FAUC468 [28] (Figure 1), demonstrating excellent tumor growth inhibition with ^177^Lu-FAUC468 (K_i_ (NTSR1) = 0.22 nM) in animal models of prostate PC3 tumor-bearing and pancreatic AsPC-1 tumor-bearing mice after the administration of single and double doses of 20–30 MBq/animal, respectively, without any detectable signs of tissue damage in the liver or kidneys [28].

The results with ^177^Lu-ligands for therapy are promising, and clinical trials have been initiated (NCT03525392). To ensure the NTSR1 expression of tumors and the individual selection of patients for such therapy trials, it would be advantageous to have readily in-house available tracers for the imaging of NTSR1-positive tumors.

Therefore, the aim of the present work was to develop and evaluate readily available ^99m^Tc-labeled NTSR1 antagonists for SPECT imaging, since Tc-99m is a unique radioisotope due to its convenient half-life (t_1/2_ = 6 h), easy availability, ideal nuclear properties, and simple preparation of ^99m^Tc-labeled radiopharmaceuticals [29]. Based on our previous work on FAUC468 [28], we applied established chelator systems of Tc-99m for the incorporation of a [^99m^Tc]Tc-HYNIC or [^99m^Tc]Tc-tricarbonyl core to give **[^99m^Tc]1** and **[^99m^Tc]2**, respectively (Figure 1). The present work deals with the optimization of the radiosynthesis of **[^99m^Tc]1** and **[^99m^Tc]2** and their in vitro and in vivo evaluation by biodistribution and SPECT/CT imaging studies in NTSR1-positive HT-29 tumor-bearing mice.

## 2. Materials and Methods

### 2.1. General Information

All chemicals were purchased from commercial sources (abcr GmbH, Karlsruhe, Germany; Thermo Fisher Scientific, Waltham, MA, USA; Carl Roth GmbH & Co. KG, Karlsruhe, Germany; Merck KGaA, Darmstadt, Germany) in the highest available quality and used without further purification. NMR spectra were acquired on a Bruker (Billerica, MA, USA) Avance Nanobay V3-I 400 MHz or a Bruker Avance III HD 600 MHz spectrometer. An (ESI) mass spectrometry analysis was performed using a Bruker Esquire 2000 instrument. High mass accuracy and resolution experiments were performed on a Bruker Daltonics timsTOF Pro spectrometer using electrospray ionization (ESI) as an ionization source. Radio-HPLC was performed on an Agilent 1100 system (Agilent Technologies Inc., Santa Clara, CA, USA). Radioactivity was detected by a HERM LB 500 radioactivity detector equipped with a NaI probe LB 6668-A (Berthold Technologies GmbH & Co. KG, Bad Wildbad, Germany). A data analysis was performed using OpenLAB CDS ChemStation Edition Software (version A.02.09, Agilent Technologies, Santa Clara, CA, USA). HPLC methods: method 1: column: Kromasil 100 C8, 5 μm, 125 × 8 mm, flow rate: 4 mL/min, solvent A: H_2_O (0.1% TFA), solvent B: MeCN (0.1% TFA), gradient A/B (%): 90:10 to 0:100 in 20 min; method 2: column: Chromolith RP-18e, 100 × 4.6 mm, flow rate: 4 mL/min, solvent A: H_2_O (0.1% TFA), solvent B: MeCN (0.1% TFA), gradient A/B (%): 90:10 to 0:100 in 10 min. 2-(1-(4-(1-(3-((3-Aminopropyl)(methyl)amino)propyl)-1H-1,2,3-triazol-4yl)-2-isopropylphenyl)-5-(2,6-dimethoxyphenyl)-1H-pyrazole-3-carboxamido)adamantane-2-carboxylic acid (**3**) was prepared as previously described [28]. Na [^99m^Tc]TcO_4_ (0.9% NaCl) was eluted from ^99^Mo/^99m^Tc generators (^99^Mo/^99m^Tc generator Ultra-Technekow and ^99^Mo/^99m^Tc generator Tekcis, Curium Pharma, London/Paris, UK/France). The elution of the ^99^Mo/^99m^Tc generators was carried out daily to avoid enrichment of technetium-99.

### 2.2. Synthesis of 2-(1-(4-(1-(3-((3-(6-(2-(Tert-butoxycarbonyl)hydrazineyl)nicotinamido)-propyl)(methyl)amino)propyl)-1H-1,2,3-triazol-4-yl)-2-isopropylphenyl)-5-(2,6-dimethoxy-phenyl)-1H-pyrazole-3-carboxamido)adamantane-2-carboxylic acid (***4***)

Compound **3** (188 mg, 254 µmol [28]) was dissolved in DMF (7 mL), followed by the addition of succinimidyl 6-(tert-butoxycarbonyl)-hydrazinopyridine-3-carboxylate (179 mg, 511 µmol [30]). The yellowish solution was stirred at room temperature overnight, and subsequently, the solvent was evaporated in vacuo. The residue was purified by automated flash column chromatography (RP18, acetonitrile/water 1:2 with 0.1% TFA) to obtain **4** (210 mg, 216 µmol, 85%) after lyophilization as a slightly yellowish solid. LCMS (ESI): *m*/*z* 974.54 [M+H]^+^ and 487.65 [M+2H]^2+^. HRMS (ESI): *m*/*z* calcd. For C_52_H_68_N_11_O_8_: 974.5247 [M+H]^+^, *m*/*z* found: 974.5258 [M+H]^+^, measured with an accuracy of 1.1 ppm and *m*/*z* calcd. For C_52_H_69_N_11_O_8_: 487.7660 [M+2H]^2+^, *m*/*z* found: 487.7661 [M+2H]^2+^, measured with an accuracy of 0.1 ppm. ^1^H NMR (DMSO-*d*_6_, 600 MHz): *d* 8.92 (s, 1H, NH), 8.65 (s, 1H, triazole-*H*), 8.52 (d, *J* = 1.8 Hz, 1H, pyridine-*H*-2), 7.94 (d, *J* = 8.6 Hz, 1H, pyridine-*H*-5), 7.80 (d, *J* = 1.8 Hz, 1H, 2-isopropylphenyl-*H*-3), 7.72–7.66 (m, 1H, pyridine-*H*-4), 7.61 (dd, *J* = 8.2, 1.9 Hz, 1H, 2-isopropylphenyl-*H*-5), 7.27 (t, *J* = 8.5 Hz, 1H, dimethoxyphenyl-*H*-4), 7.20 (d, *J* = 8.2 Hz, 1H, isopropylphenyl-*H*-6), 6.87 (s, 1H, pyrazole-*H*), 6.59 (d, *J* = 9.3 Hz, 2H, dimethoxyphenyl-*H*-3 and 5), 4.41 (t, *J* = 6.9 Hz, 2H, triazole-C*H*_2_CH_2_CH_2_), 3.64 (s, 6H, OC*H*_3_), 3.45–3.25 (m, 6H, C*H*_2_), 2.67 (sept, *J* = 6.7 Hz, 1H, isopropyl-C*H*), 2.62–2.59 (m, 2H, C*H*_2_), 2.31 (dt, *J* = 14.0, 6.9 Hz, 2H, CH_2_C*H*_2_CH_2_), 2.19–2.17 (m, 2H, adamantyl-*H*), 2.14 (s, 3H, NC*H*_3_), 2.02–1.94 (m, 2H, adamantly-*H*), 1.71–1.54 (m, 10H, adamantyl-*H*), 1.66 (s, 9H, Boc-*H*), 1.08 (d, *J* = 5.7 Hz, 6H, isopropyl-C*H*_3_) ppm. ^13^C NMR (DMSO-*d*_6_, 150 MHz): *d* 174.5, 166.9, 164.8, 161.5, 159.6, 158.0, 155.8, 147.7, 147.4, 146.2, 145.5, 138.7, 136.5, 131.7, 131.5, 131.5, 128.6, 127.7, 122.7, 122.2, 121.9, 120.6, 107.9, 106.5, 103.7, 79.1, 55.6, 54.9, 53.8, 47.8, 41.6, 37.8, 37.5, 34.1, 33.2, 32.6, 28.1, 27.5, 27.3, 26.9, 26.8, 26.5, 24.6, 22.5 ppm.

### 2.3. Synthesis of 2-(5-(2,6-Dimethoxyphenyl)-1-(4-(1-(3-((3-(6-hydrazineylnicotinamido)propyl)-(methyl)amino)propyl)-1H-1,2,3-triazol-4-yl)-2-isopropylphenyl)-1H-pyrazole-3-carboxamido)-adamantane-2-carboxylic Acid (***5***)

Compound **4** (7 mg, 7.26 µmol) was dissolved in TFA (400 µmol), and the yellow solution was stirred at room temperature for 15 min. Thereafter, a mixture of acetonitrile (0.1% TFA, 300 µL) and water (0.1% TFA, 300 µL) were added, and the crude product in solution was purified by semi-preparative HPLC (method 1, *t*_R_ = 9.18 min). Product **5** (3.17 mg, 3.63 µmol, 50%) was obtained after lyophilization as a white solid. HPLC: *t*_R_ = 4.11 min (method 2), *t*_R_ = 9.18 min (method 1), purity > 99%. LCMS (ESI): *m*/*z* 874.45 [M+H]^+^ and 437.60 [M+2H]^2+^. HRMS (ESI): *m*/*z* calcd. For C_47_H_60_N_11_O_6_: 874.4723 [M+H]^+^, *m*/*z* found: 874.4722 [M+H]^+^, measured with an accuracy of 0.1 ppm and *m*/*z* calcd. For C_47_H_61_N_11_O_6_: 437.7398 [M+2H]^2+^, *m*/*z* found: 437.7402 [M+2H]^2+^, measured with an accuracy of 0.9 ppm. ^1^H NMR (DMSO-*d*_6_, 600 MHz): *d* 8.65 (s, 1H, triazole-*H*), 8.58 (d, *J* = 1.5 Hz, 1H, pyridine-*H*-2), 8.09 (dd, *J* = 8.9, 2.1 Hz, 1H, pyridine-*H*-4), 7.81 (d, *J* = 1.9 Hz, 1H, 2-isopropylphenyl-*H*-3), 7.62 (dd, *J* = 8.2, 1.8 Hz, 1H, 2-isopropylphenyl-*H*-5), 7.36 (s, 1H, N*H*), 7.28 (t, *J* = 8.4 Hz, 1H, dimethoxyphenyl-*H*-4), 7.24 (d, *J* = 8.2 Hz, 1H, isopropylphenyl-*H*-6), 6.89 (d, *J* = 8.9 Hz, 1H, pyridine-*H*-5), 6.71 (s, 1H, pyrazole-*H*), 6.61 (d, *J* = 8.5 Hz, 2H, dimethoxyphenyl-*H*-3 and 5), 4.50 (t, *J* = 6.7 Hz, 2H, triazole-C*H*_2_CH_2_CH_2_), 3.65 (s, 6H, OC*H*_3_), 3.32–3.07 (m, 6H, C*H*_2_), 2.78 (s, 3H, NC*H*_3_), 2.67 (sept, *J* = 6.8 Hz, 1H, isopropyl-C*H*), 2.54 (s, 2H, CH_2_C*H*_2_CH_2_), 2.33–2.20 (m, 2H, CH_2_C*H*_2_CH_2_), 2.10–1.62 (m, 14H, adamantyl-*H*), 2.14 (s, 3H, C*H*_3_), 1.09 (d, *J* = 5.6 Hz, 6H, isopropyl-C*H*_3_) ppm. ^13^C NMR (DMSO-*d*_6_, 150 MHz): *d* 173.3, 160.2, 158.1, 157.9, 157.7, 146.3, 146.2, 145.7, 139.0, 138.9, 137.9, 137.9, 136.5, 131.6, 127.7, 122.7, 122.2, 122.2, 108.8, 108.2, 106.3, 103.7, 62.2, 59.2, 55.6, 53.3, 52.5, 46.8, 37.3, 36.3, 36.1, 33.4, 32.6, 32.0, 27.4, 26.3, 26.1, 24.3, 24.1 ppm.

### 2.4. Synthesis of 2-(1-(4-(1-(3-((3-(3-(4-(2-Amino-2-carboxyethyl)-1H-1,2,3-triazol-1-yl)-propanamido)propyl)(methyl)amino)propyl)-1H-1,2,3-triazol-4-yl)-2-isopropylphenyl)-5-(2,6-dimethoxyphenyl)-1H-pyrazole-3-carboxamido)adamantane-2-carboxylic Acid (***6***)

A solution of **3** (90 mg, 122 µmol) in DMF (7 mL) and *N,N*-diisopropylethylamine (1 mL) was cooled to 0 °C, and 3-azidopropanoate-*N*-hydroxysuccinimide ester (25.8 mg, 122 µmol) was added to the solution. After a 1 h stirring time, the reaction solution was allowed to warm to room temperature. Subsequently, propargylglycine (13.8 mg, 122 µmol, racemic mixture), Cu(II) sulfate pentahydrate (30.4 mg, 122 µmol) in water (3 mL), and sodium ascorbate (48.8 mg, 246 µmol) in water (3 mL) were added, and the reaction mixture was stirred at room temperature for 30 min. Thereafter, the solvent was evaporated in vacuo, and the residue was purified by automated flash column chromatography (RP18, acetonitrile/water 2:1 with 0.1% TFA) to obtain **6** (30.6 mg, 33.3 µmol, 27%) after lyophilization as a white solid. HPLC: t_R_ = 4.13 min (method 2), purity > 99%. LCMS (ESI): *m*/*z* 949.48 [M+H]^+^ and 475.36 [M+2H]^2+^. HRMS (ESI): *m*/*z* calcd. For C_49_H_66_N_12_O_8_: 475.2558 [M+2H]^2+^, *m*/*z* found: 475.2561 [M+2H]^2+^, measured with an accuracy of 0.7 ppm.

### 2.5. Synthesis of **[^nat^Re]2**

Compound **6** (3 mg, 3.16 μmol) was dissolved in dry methanol, and DIPEA (2 μL, 11.7 μL) was subsequently added to the solution. After 5 min, ReBr(CO)_5_ (1.28 mg, 3.16 μmol) was added, and the reaction solution was stirred at 60 °C overnight. The crude product in solution was purified by semi-preparative HPLC (method 1, t_R_ = 10.1 min). Product **[^nat^Re]2** (1.96 mg, 1.61 μmol, 51%) was obtained after lyophilization as a white solid. HPLC: t_R_ = 4.32 min (method 2), t_R_ = 10.1 min (method 1), purity > 99%. LCMS (ESI): *m*/*z* 610.84 [M+2H]^+^. HRMS (ESI): *m*/*z* calcd. For C_52_H_65_N_12_O_11_Re: 610.2221 [M+2H]^+^, *m*/*z* found: 610.2219 [M+2H]^+^, measured with an accuracy of 0.6 ppm.

### 2.6. Radiosynthesis of **[^99m^Tc]1**

The radiosynthesis of **[^99m^Tc]1** followed the known procedure of ^99m^Tc-labeling of HYNIC precursors [31,32,33,34,35]. [^99m^Tc]TcO_4_^−^ (1.04 GBq) in NaCl solution (0.9%, 450 µL) was added to a mixture of **5** (1 nmol) in water (10 µL), tin(ll) chloride (c = 1 mg/mL) in aqueous HCl (0.2 M, 20 µL), sodium hydroxide (0.1 M, 50 µL), and tricine (c = 70 mg/mL, 500 µL). The pH was determined to be 6–7, and the reaction was heated at 60 °C for 30 min. Subsequently, the reaction solution was diluted with water (15 mL) and was passed through a preconditioned (acetonitrile and water, 5 mL each) Sep-Pak^®^ light tC18 cartridge. The cartridge was washed with water (2 mL), followed by elution with ethanol (2 mL) and evaporation of the solvent in vacuo. **[^99m^Tc]1** was reconstituted with PBS for further experiments or with a cell culture medium for the cellular uptake experiments. Starting from 1 GBq of [^99m^Tc]TcO_4_^−^, this procedure yielded 750 MBq of **[^99m^Tc]1** (94% RCY, 75% RAY) after a total synthesis time of 40 min. HPLC: *t*_R_ = 3.96 min (method 2), *t*_R_ = 8.43 min (method 1), radiochemical purity: 96%.

### 2.7. Radiosynthesis of **[^99m^Tc]2**

The radiosynthesis of **[^99m^Tc]2** followed the previously reported “click-to-chelate” method [36,37]. [^99m^Tc]TcO_4_^−^ (1.1 GBq) in NaCl solution (0.9%, 1 mL) was added to a CRS Kit (Paul Scherrer Institute, Villigen, Switzerland), and the reaction solution was heated at 100 °C for 30 min to afford the tricarbonyl precursor [^99m^Tc][Tc(CO_3_)(H_2_O)]^+^ as an intermediate. Thereafter, the pH value was set to 7 by addition of an acidified phosphate buffer solution (NaH_2_PO_4_/Na_2_HPO_4_ (1 M, 1:1) and HCl (1 M) (1:2), 250 µL). Compound **6** (20 nmol) in water (20 µL) was added, and the reaction mixture was heated at 100 °C for a further 30 min. Afterwards, the product **[^99m^Tc]2** was isolated by semi-preparative HPLC (method 1, *t*_R_ = 11.5 min). The product fraction was diluted with water (15 mL), and the solution was passed through a preconditioned (acetonitrile and H_2_O, 5 mL each) Sep-Pak^®^ light tC18 cartridge. The cartridge was eluted with ethanol (2 mL), and the solvent was evaporated in vacuo. **[^99m^Tc]2** was reconstituted with PBS for further experiments or with a cell culture medium for cellular uptake experiments. Starting from 1.1 GBq of [^99m^Tc]TcO_4_^−^, this procedure yielded 684 MBq **[^99m^Tc]2** (96% RCY, 62% RAY) after a total synthesis time of 80 min. HPLC: *t*_R_ = 4.91 min (method 2), *t*_R_ = 11.5 min (method 1), radiochemical purity: 99%.

### 2.8. In Vitro Characterization of Radiotracers

#### 2.8.1. Lipophilicity Determination

The lipophilicity of the radioligands was determined by the distribution coefficient log *D*_7.4_. The respective radioligand (10 µL, about 25 kBq in 10 µL PBS) was added to a mixture of PBS (500 µL) and 1-octanol (500 µL), and the emulsion was vortexed for 1 min. After centrifugation, three samples of 100 µL each were taken from each layer and analyzed by a γ-counter. The partition coefficient was calculated as log[cpm (octanol)/cpm (PBS)], and the data were expressed as mean values ± SD from three experiments.

#### 2.8.2. Determination of Plasma Protein Binding

The binding of ^99m^Tc-labeled compounds to plasma proteins was determined using gel filtration columns (illustra^™^ MicroSpin^™^ G-50 Columns, GE Healthcare Life Sciences, Freiburg, Germany). An aliquot of the radiotracer (approx. 100 kBq in 10 µL PBS) was added to 100 µL of PBS and 100 µL of human plasma, respectively. Both samples were incubated at 37 °C for 10 min. MicroSpin^™^ columns were prepared according to the user instruction. Then, 40 µL of the incubated radiotracer was added onto the columns and the devices were centrifuged (2000× *g*, 2 min). The radioactivity eluted from the resin was calculated as a percentage of the total amount of radioactivity. The sample in saline was used as a control.

#### 2.8.3. Determination of Radiotracer Stability in Human Serum and Human Plasma

The stability of ^99m^Tc-labeled compounds was determined in human serum and human plasma. An aliquot of the radiotracer (5–10 MBq in 10 µL PBS) was added to 200 µL of human serum/plasma and incubated at 37 °C. Aliquots of 20 µL were taken after 10, 30, 60, 120, 240, and 360 min and quenched in 20 µL MeOH. The samples were centrifuged (20,000× *g*, 2 min), and the supernatants were analyzed by radio-HPLC (Chromolith RP-18e, 100 × 4.6 mm, 4 mL/min, 0–10 min 10–100% CH_3_CN (0.1% TFA) in water (0.1% TFA).

#### 2.8.4. Cell Culture

The HT-29 cell line was purchased from Cell Lines Service GmbH (Eppelheim, Germany). The HT-29 cells were cultivated in Minimum Essential Medium Eagle with Earle’s salts (Sigma-Aldrich, St. Louis, MO, USA) and supplemented with 10% fetal calf serum (FCS), 1% *L*-glutamine, 1% non-essential amino acids (NEAA), and 1% pyruvate. All cells were cultured under sterile conditions in a humidified atmosphere containing 5% CO_2_ at 37 °C. Cells were routinely passaged twice a week.

#### 2.8.5. Competitive Cellular Uptake Assay

Compounds **[^99m^Tc]1** and **[^99m^Tc]2** were used as radioactive test compounds, and FAUC468 was used as the non-radioactive competitor in cellular uptake experiments using NTSR1-positve HT-29 cells. For the determination of the IC*_50_* values, 8–10 concentrations of FAUC468 were used. The HT-29 cells were seeded on the day of the experiment in a 96-well-plate (~500.000 cells per well in 200 µL cell medium) and were incubated in a humidified atmosphere at 37 °C containing 5% CO_2_. Subsequently, the 96-well-plate was centrifuged at 850 rpm for 2 min following aspiration of the cell medium. The cells were washed with cell medium (200 µL), the plate was centrifuged at 850 rpm for 2 min, and the supernatant was aspirated. The incubation solutions (0.009–900 nM FAUC468 in 90 µL cell medium per well) and the respective radiotracer (10 µL, 100 kBq per well) were given to the wells, and the plates were incubated at 37 °C for 1 h. After the incubation time, the plate was centrifuged at 850 rpm for 2 min. The supernatant was removed, followed by two washing steps with the cell medium (200 µL) and PBS (200 µL). Sodium hydroxide solution (200 µL, 2 M, 37 °C) was added, and after 7 min, solutions of the cell homogenates were transferred into tubes, which were measured for radioactivity in a γ-counter (Wallac Wizard, Perkin Elmer, Waltham, MA, USA). The IC_50_ values of the competition curves from the radioligand uptake experiments were accomplished by a non-linear regression analysis using the algorithms (one site homologous for FAUC468 and one site heterologous with depletion for the respective radiotracer) in PRISM 5.0 (GraphPad Software, San Diego, CA, USA). Three independent experiments were performed for each ^99m^Tc-ligand, with each FAUC468 concentration used in triplicates.

#### 2.8.6. Cellular Uptake Assay

The cellular uptake of the respective radiotracer was determined after 30 min, 1 h, 2 h, and 4 h. Nonspecific uptake was determined by coincubation with FAUC468 (1 µM). The HT-29 cells were seeded in 24-well-plates one day before the experiment (~500.000 cells per well in 200 µL of the cell culture medium). The cells were incubated under sterile conditions in a humidified atmosphere at 37 °C containing 5% CO_2_. On the day of the experiment, the cells were washed with the cell culture medium (1 mL) and with PBS (500 mL, 2×). The respective radiotracer (in 500 µL cell medium, 100 kBq per well) was added to the wells. Nonspecific cellular uptake was measured in the presence of FAUC468 (1 µM). After the respective incubation time (37 °C), the incubation solutions were aspirated, and the cells were washed with ice-cold PBS (500 µL, 3×). Afterwards, sodium hydroxide solution (500 µL, 0.1 M, 37 °C) was added, and after 7 min, the solutions of cell homogenates were transferred into tubes, which were measured in a γ-counter (Wallac Wizard, Perkin Elmer, Waltham, MA, USA). The protein concentration of each well was determined in duplicates using the Bradford reagent (Bio-Rad Laboratories GmbH, Feldkirchen, Germany). A data analysis was performed using the software PRISM 5.0 (GraphPad Software, San Diego, CA, USA).

#### 2.8.7. Receptor Binding Assays

Radioligand binding experiments with the NTSR1 and NTSR2 receptor subtypes were performed as described previously [38,39]. In detail, NTSR1 competition binding was measured using homogenates of membranes from CHO cells stably expressing the human NTSR1 [40], with 1–4 μg protein/well and the radioligand [^3^H]neurotensin (specific activity 101 Ci/mmol; PerkinElmer, Rodgau, Germany) at a final concentration of 0.50 nM. Specific binding of the radioligand was determined to have a K*_D_* value of 0.83 ± 0.20 nM and a B_max_ of 5500 ± 840 fmol/mg protein, respectively. NTSR2 binding was measured using homogenates of membranes from HEK 293T cells, which were transiently transfected with DNA encoding the human NTSR2 (cDNA Resource Center, Bloomsberg, PA, USA) using the Mirus TransIT-293 transfection reagent (Peqlab, Erlangen, Germany). Membranes were incubated at a final concentration of 6210 μg protein/well together with 0.50 nM of [^3^H]NT(8-13) (specific activity 136 Ci/mmol; custom synthesis of [leucine-^3^H]NT(8-13) by GE Healthcare, Freiburg, Germany) at a K*_D_* value of 1.2 ± 0.12 nM and a B_max_ of 530 ± 64 fmol/mg protein. Nonspecific binding was determined in the presence of 10 μM NT(8-13), protein concentration was determined by the method of Lowry using bovine serum albumin as a standard [41]. A data analysis of the radioligand displacement curves was accomplished by non-linear regression analysis using the algorithms in PRISM 9.0 (GraphPad Software, San Diego, CA, USA). EC_50_ values derived from the resulting dose response curves were transformed into the corresponding K_i_ values applying the equation of Cheng and Prusoff [42].

### 2.9. In Vivo Characterization of Radiotracers

#### 2.9.1. Animal Model

All mouse experiments were approved by the local animal protection authorities (Government of Lower Franconia, Germany, No. 55.2 2532-2-279, date of approval: 16.08.2016) and performed at the FAU in accordance with the relevant E.U. guidelines and regulations. Female nude mice (~8 weeks old, Crl:NMRI-*Foxn1^nu^*, Charles River Laboratories Inc., Wilmington, MA, USA) were used for the biodistribution and SPECT/CT studies (40 mice in total), and four NMRI mice (~8 weeks old, Crl:NMRI(Han), Charles River Laboratories Inc., Wilmington, MA, USA) were used for the determination of radiotracer stability in mouse blood. There were no animal exclusions from the data analysis. The mice were kept in groups under pathogen-free conditions in an IVC recovery unit (25 °C ± 1 °C, Tecniplast S.p.A, Buggugiate, Italy) with autoclaved bedding, food, and water on a daily 12-h light/dark cycle. For the biodistribution and SPECT/CT studies, aliquots of HT-29 cells (1.5–2 × 10^6^ in PBS, 100 µL) were subcutaneously injected in female nude mice under anesthesia (O_2_/isoflurane (2–3% isoflurane), 0.8 L/min O_2_, 3–5 min). The cell suspensions were resuspended vigorously with a syringe and were injected subcutaneously in between the shoulders on the back of the animals. The mice were weighed, and the tumor sizes were measured with a caliper 3 times a week. Approximately 14 days after cell inoculation, the tumors reached a diameter of approximately 7–9 mm (corresponding to a volume of 180–380 mm^3^) when the mice were randomly assigned to the different experimental groups.

#### 2.9.2. Biodistribution

Biodistribution studies were conducted using HT-29-xenografted female nude mice (total number of animals: n = 32). On the day of the experiment, 1–3 MBq of the respective radiotracer in PBS (100 µL) were injected via the tail vein under deep isoflurane anesthesia. Afterwards, the mice were transferred to a cage and allowed to recover from the anesthesia. The mice were sacrificed by cervical dislocation under anesthesia at 1, 2, or 4 h p.i. (n = 4 each for **[^99m^Tc]1** and **[^99m^Tc]2**). The specificity of tumor uptake at 4 h p.i. was determined by pretreatment of the mice with FAUC468 (n = 4 each for **[^99m^Tc]1** and **[^99m^Tc]2**). FAUC468 (100 µg) in PBS (100 µL) was injected in the anesthetized mice via their tail veins 15 min before radioligand injection. Blood, as well as the organs/tissues—the lungs, liver, kidneys, heart, spleen, brain, muscle, femur, HT-29 tumor, intestine, pancreas, and duodenum—were harvested and analyzed in the γ-counter. The samples were weighed, and radioactivity in the different tissues was calculated as a percentage of the total injected dose per gram tissue (%ID/g).

#### 2.9.3. Small Animal SPECT/CT

Small animal SPECT/CT images were conducted on an Inveon Multimodality scanner (SPECT and CT, Siemens AG, Berlin/Munich, Germany). The SPECT/CT images were evaluated using the Inveon Acquisition Workplace software (version 2.0.0.1050) and Inveon Research Workplace software (version 4.2, Siemens Medical Solutions USA Inc., Knoxville, TN, USA). For image reconstruction, raw data from the SPECT/CT were exported as DICOM images and imported in an open-source medical imaging viewer (Horos Version 3.2.1; www.horosproject.org). Using the 2D viewer, axial CT images were overlaid with their respective SPECT data using a color code ranging from black (low values) to white (high values). Furthermore, a 3D volume rendering of the SPECT/CT data was performed with the same settings. Small animal SPECT/CT studies were conducted using HT-29-xenografted female nude mice (total number of animals: n = 8, four mice per radiotracer). In total, 60–110 MBq of the respective radiotracer in PBS (100 µL) were injected via the tail vein under deep isoflurane anesthesia. Afterwards, the mice were transferred to a cage and allowed to recover from the anesthesia. At 1 h, 4 h, and 24 h p.i., the mice were anesthetized, transferred, and positioned for the SPECT/CT scan, so that the complete torso was apparently inside the field of view. Static SPECT images were acquired for 18 min and CT images for 8 min, and the anesthetized mice were tempered with a heating pad (37 °C). After each scan, the mice were allowed to recover from the anesthesia in their cage. The same procedure was applied for blocking experiments using the same mice 2 days later. For these experiments, FAUC468 (100 µg) in PBS (100 µL) was preinjected via the tail vein 15 min before the radiotracer injection. After the experiments, the mice were euthanized by cervical dislocation under deep isoflurane anesthesia.

#### 2.9.4. Determination of Radiotracer Stability in Mouse Blood

NMRI mice (n = 4, approximately 8 weeks old, Crl:NMRI(Han), Charles River Laboratories Inc., Wilmington, MA, USA) were anesthetized with O_2_/isoflurane (2–3% isoflurane, 0.8 L/min O_2_, 3–5 min), and the respective radiotracer was injected via the tail vein (3–12 MBq in PBS (100 µL)/mouse, one mouse per time point). After 30 and 60 min p.i., the animals were sacrificed by cervical dislocation under deep isoflurane anesthesia. The blood samples were transferred into heparin-coated Microvette^®^ tubes and centrifuged at 2000 rpm for 2 min. The blood plasma (supernatant, 30 μL) was collected, and acidified water (30 μL, 10% TFA) was added for denaturation of the plasma proteins. The samples were centrifuged at 2000 rpm, and the supernatant (50 μL) was analyzed by radio-HPLC (Chromolith RP-18e, 100 × 4.6 mm, 4 mL/min, 0–10 min 10–100% CH_3_CN (0.1% TFA) in water (0.1% TFA) to determine the percent of intact radiotracer.

### 2.10. Statistics

A statistical analysis of the biodistribution and SPECT results was performed using an ordinary Two-Way ANOVA and a two-tailed, *t*-test, respectively, with PRISM 9.0 (GraphPad Software, San Diego, CA, USA). The required minimum number (n = 4) of animals per group to determine tracer specificity is sufficient to demonstrate that reduced tracer uptake by at least 50% is statistically significant (SP > 0.8, α-error: 0.05, mean values with SD of 25%). A difference was considered significant at a probability value of *p* ≤ 0.05.

## 3. Results

### 3.1. Chemistry and Radiochemistry

**Scheme 1:**
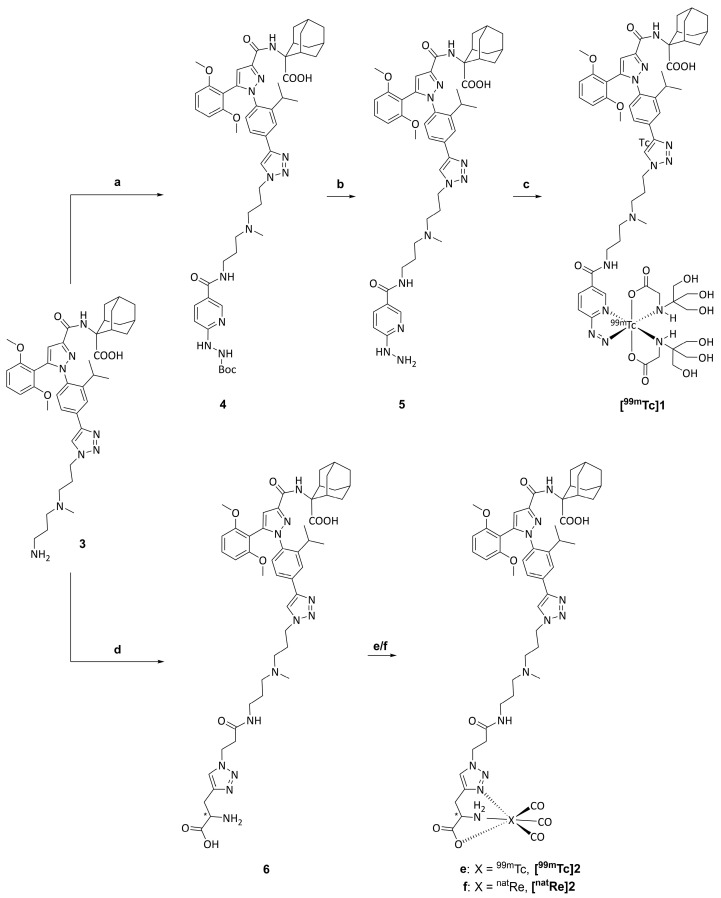
Radiosynthesis of HYNIC complex **[^99m^Tc]1** and [^99m^Tc]Tc-tricarbonyl complex **[^99m^Tc]2**. Reaction conditions: (**a**) succinimidyl 6-(*tert*-butoxycarbonyl)-hydrazinopyridine-3-carboxylate [30], DMF, rt, o/n, 85%; (**b**) TFA, rt, 15 min, 50%; (**c**) [^99m^Tc]TcO_4_^−^ SnCl_2_, tricine, NaCl (0.9%), pH = 7, 60 °C, 30 min, RCY = 94%, RAY = 75%, RCP = 96%; (**d**) 1. 3-azidopropanoate-N-hydroxysuccinimide ester, DMF, DIPEA, 0 °C, 1 h; 2. propargylglycine, CuSO_4_, sodium ascorbate, DMF, H_2_O, 30 min, rt, 27%, (**e**) [^99m^Tc][Tc(CO)_3_(H_2_O)_3_]^+^ (CRS kit), NaCl (0.9%), pH = 7, 100 °C, 30 min, RCY = 96%, RAY = 62%, RCP ≥ 99%; (**f**) ReBr(CO)_5_, DIPEA, MeOH, 60 °C, o/n.

The course of the synthesis of amine **3** was performed as described previously [28]. Starting from **3**, two new precursors (**5** and **6**) were synthesized using the ^99m^Tc-chelator systems hydrazinonicotinamide (HYNIC) and *DL*-propargylglycine, respectively, which allow for two different radiolabeling strategies to be applied, namely the HYNIC [34] and the click-to-chelate approach [37], for the incorporation of a [^99m^Tc]Tc-HYNIC (**[^99m^Tc]1**) and a [^99m^Tc]Tc-tricarbonyl core (**[^99m^Tc]2**) (Scheme 1).

The radiosynthesis of the [^99m^Tc]Tc-HYNIC complex **[^99m^Tc]1** was performed with tricine as a co-ligand and optimized with respect to the minimum amount of required precursor. The HYNIC precursor **5** was added to a solution of tricine and tin(II) chloride, and the pH was adjusted to 7 with NaOH solution. [^99m^Tc]TcO_4_^−^ was added to the reaction mixture in a total volume of 1030 µL, followed by heating at 60 °C for 30 min. For the radiosynthesis of **[^99m^Tc]1**, only 1 nmol (0.9 µg) of HYNIC precursor **5** was required, which is remarkable, as 5–20 µg of the respective HYNIC precursor is typically used [31,32,33,35]. **[^99m^Tc]1** was obtained in a high radiochemical yield (RCY) of 94%, thus without the need for further purification, and in excellent molar activity (A_m_) of 1000 GBq/µmol, due to the low amount of precursor **5** in the final solution. In order to separate **[^99m^Tc]1** from tricine and reductive tin salts, **[^99m^Tc]1** was purified by solid-phase extraction (SPE) for further in vitro and in vivo experiments, marginally increasing the RCP to 96% and achieving finally formulated **[^99m^Tc]1** in a total radioactivity yield (RAY) of 75%. Notably, the reaction turned out to be sensitive to the formation of an unknown radioactive by-product, which was dependent on the concentration of precursor **5**. To achieve a more robust Kit-like ^99m^Tc-labeling process, we developed a modified composition of a lyophilisate, consisting of all components for direct labeling with a [^99m^Tc]pertechnetate eluate, however, these lyophilisates were not used in the current study.

For the radiosynthesis of the ^99m^Tc-tricarbonyl-labeled NTSR1 ligand **[^99m^Tc]2**, the complex [^99m^Tc][Tc(CO)_3_(H_2_O)_3_]^+^ was synthesized in a first step by incubation of [^99m^Tc][TcO_4_]^−^ with a CO-donor salt under reductive reaction conditions at 100 °C for 30 min, applying the CRS kit system (Center for Radiopharmaceutical Sciences, Paul-Scherrer-Institut, Villigen, Switzerland). In the second step, the pH of the reaction solution (pH > 10) was adjusted to 7 with an acidified phosphate buffer, as reported by Mindt and co-workers [36]. The precursor **6** was added to the reaction mixture and the vial was heated at 100 °C for a further 30 min. **[^99m^Tc]2** was obtained in a RCY of 96% and was further purified by HPLC to provide an excellent RCP (>99%) and a molar radioactivity of 55 GBq/µmol.

### 3.2. In Vitro Characterization of Radiotracers

The distribution coefficient values (log *D*_7.4_) of radioligands **[^99m^Tc]1** and **[^99m^Tc]2** were determined by the shake-flask method using 1-octanol and PBS (pH = 7.4) and reported in Table 1. Radioligand **[^99m^Tc]2** (log *D*_7.4_ = 1.0) with the ^99m^Tc-tricarbonyl core revealed moderate hydrophilicity as the glycine moiety is involved in the complexation of Tc-99m. The relatively low hydrophilicity of **[^99m^Tc]2** was in accordance with the observation by an HPLC analysis, where significantly different retention times of the labeling precursor (**6**) (4.13 min) and the radioligand (**[^99m^Tc]2**) (4.91 min) were detected. In addition, the 1,2,3-triazole moiety contributes to the lipophilicity. Contrarily, the ^99m^Tc-HYNIC complex **[^99m^Tc]1** showed significantly higher hydrophilicity with the log *D*_7.4_ value of −0.27, due to the hydroxyl-containing tricine co-ligands.

The plasma protein binding (PPB) of **[^99m^Tc]1** and **[^99m^Tc]2** was determined by incubation with human plasma and subsequent gel filtration. In parallel to the differences in lipophilicity, **[^99m^Tc]2** showed higher PPB compared to **[^99m^Tc]1**, suggesting a higher free fraction of **[^99m^Tc]1** in the blood after tracer injection. The control experiment using PBS instead of plasma revealed >99% of free **[^99m^Tc]1** and 99% of free **[^99m^Tc]2**.

The stability of **[^99m^Tc]1** and **[^99m^Tc]2** was determined in human serum and human plasma. Both tracers showed high stability of at least 73% of the intact tracers after long-term 6 h incubation in the serum, as determined by radio-HPLC (Table 1).

**Figure 2:**
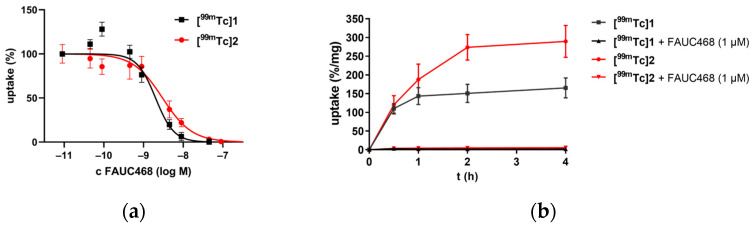
In vitro characterization of **[^99m^Tc]1** and **[^99m^Tc]2**. (**a**) Competitive NTSR1-mediated uptake (binding and internalization) of **[^99m^Tc]1** and **[^99m^Tc]2** in HT-29 cells in the presence of increasing concentrations of FAUC468. The cellular radioactivity was normalized to the value at lowest concentration of FAUC468. Data are expressed as mean values of cellular uptake (%) ± SD from three independent experiments, each performed in triplicate. IC_50_ (**[^99m^Tc]1)** = 1.93 nM, IC_50_ (**[^99m^Tc]2**) = 3.12 nM. (**b**) Time dependency of cellular uptake of **[^99m^Tc]1** and **[^99m^Tc]2** in HT-29 cells. Nonspecific cellular uptake was determined by coincubation with FAUC468 (1 µM). Data are expressed as mean values of cellular uptake (%/mg) ± SD from three independent experiments, each performed in quadruplicate.

The half maximal inhibitory constant (IC_50_) was determined for FAUC468 by competitive uptake experiments with **[^99m^Tc]1** and **[^99m^Tc]2** (Figure 2a). As the non-radioactive ^99^Tc-analogs were not available and the synthesis of the Re-HYNIC complex as a surrogate of **[^99m^Tc]1** was unsuccessful, we determined the binding affinities of **[^99m^Tc]1** and **[^99m^Tc]2** by competitive binding experiments with each ^99m^Tc-ligand using increasing concentrations of the known NTSR1 ligand FAUC468 (K_i_ = 1.9 nM, Table 2) on adherent HT-29 cells. The mean IC_50_ values of FAUC468 were 1.93 nM for the displacement of **[^99m^Tc]1** and 3.12 nM for the displacement of **[^99m^Tc]2** (Figure 2a). Hence, **[^99m^Tc]2** exhibited a higher affinity for NTSR1 than **[^99m^Tc]1**.

The results on the IC_50_ values of FAUC468 for the displacement of **[^99m^Tc]1** and **[^99m^Tc]2** were in good agreement with the K_i_ values for NTSR1 and NTSR2 determined for the synthesized ligands **5** (HYNIC precursor of **[^99m^Tc]1**), **6** (glycine precursor of **[^99m^Tc]2**), and **[^nat^Re]2** (structural analog of **[^99m^Tc]2**), which are listed in Table 2.

Interestingly, the K_i_ values for the NTSR1 and NTSR2 of FAUC468 and **[^nat^Re]2** were very similar (Table 2). Given the similarity of the IC_50_ values for FAUC468 in displacement studies with **[^99m^Tc]1** and **[^99m^Tc]2** (Figure 2a), we conclude that **[^99m^Tc]1** would have a K_i_ value for NTSR1 and NTSR2 marginally higher than that of FAUC468. The HYNIC-precursor of **[^99m^Tc]1**, compound **5**, revealed a 5-fold lower NTSR1 affinity compared to FAUC468 and **[^nat^Re]2**, whereas the triazolyl glycine derivative **6** surprisingly showed subnanomolar affinity with a K_i_ value of 0.3 nM (Table 2). It is therefore tempting to speculate that solvent hydrogen bond interactions with the glycine moiety of **6** positively influence the binding affinity. Due to the similar K_i_ values of FAUC468 and the Re-tricarbonyl complex **[^nat^Re]2** (Table 2), we expect similar K_i_ values for **[^99m^Tc]1** and **[^99m^Tc]2** in the range of 2 nM, with a slightly higher NTSR1 affinity of **[^99m^Tc]2**, as shown by the comparative displacement studies with FAUC468 (Figure 2a).

In radioligand uptake assays using HT-29 cells, the ^99m^Tc-tricarbonyl complex **[^99m^Tc]2** showed high cellular uptake with maximum values of 250–300%/mg at 2 to 4 h (Figure 2b). A similar time course was determined for the ^99m^Tc-HYNIC complex **[^99m^Tc]1**. However, the uptake after 2 and 4 h was about half than for **[^99m^Tc]1** (120–150%/mg, Figure 2b). These results confirmed the higher NTSR1 affinity of **[^99m^Tc]2** compared to **[^99m^Tc]1**, which is consistent with the competitive cellular uptake studies (Figure 2a), in which a slightly higher IC_50_ of FAUC468 was determined for the displacement of **[^99m^Tc]2** than for **[^99m^Tc]1**. The specificity of NTSR1-mediated cellular uptake of the radioligands binding was proven by blocking the uptake in the presence of FAUC468 (1 µM), as only negligible cellular uptake was observed for both radioligands at all time points during tracer incubation (Figure 2b).

### 3.3. In Vivo Characterization of Radiotracers

**Figure 3:**
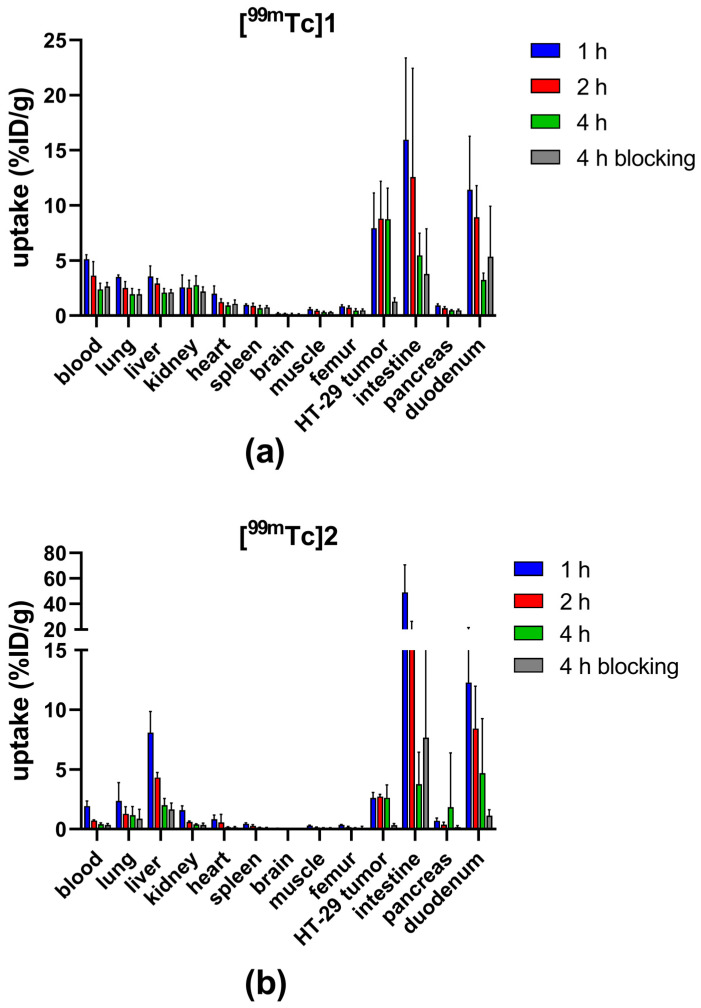
(**a**) Biodistribution study of **[^99m^Tc]1** in HT-29 tumor-bearing nude mice. (**b**) Biodistribution study of **[^99m^Tc]2** in HT-29 tumor-bearing nude mice. Pretreatment of animals with FAUC468 (100 µg/mouse) was performed 15 min prior to tracer injection (“blocking”). Data were expressed as mean values (%ID/g) ± SD at 1, 2, and 4 h p.i. (n = 4 per group).

The in vivo properties of the ^99m^Tc-HYNIC (**[^99m^Tc]1**) and ^99m^Tc-tricarbonyl (**[^99m^Tc]2**) complexes were analyzed in biodistribution studies using HT-29 tumor-bearing nude mice (Figure 3). A comprehensive statistical analysis comparing **[^99m^Tc]1** and **[^99m^Tc]2** for all time points post-injection (p.i.) is provided in Figure S1 (see the Supporting Information). The ^99m^Tc-tricarbonyl complex **[^99m^Tc]2** (log *D*_7.4_ = 1.0) was known to be more lipophilic than the ^99m^Tc-HYNIC complex **[^99m^Tc]1** (log *D*_7.4_ = −0.27), which was confirmed by the results of the biodistribution studies: The radioligand **[^99m^Tc]1** showed higher tissue uptake than **[^99m^Tc]2** in almost every organ (Supporting Information Figure S1). The tumor uptake for **[^99m^Tc]1** was with 8.8 ± 3.4 %ID/g at 2 h p.i. higher than that of **[^99m^Tc]2** (2.7 ± 0.2 %ID/g, 2 h p.i.). Moreover, the tumor retention was high for both tracers, showing no wash-out from 1 to 4 h p.i. **[^99m^Tc]2** was rapidly cleared from blood circulation over time and showed high initial liver uptake, whereas **[^99m^Tc]1** revealed still 2.2 ± 0.05 %ID/g at 4 h p.i. in blood, although being more hydrophilic with lower plasma protein binding in vitro than **[^99m^Tc]2** (Table 1). Moreover, the stability of the radioligands in vivo was evaluated using NMRI mice, which demonstrated that approximately 65% of the intact radioligand was present in blood at 30 and 60 min p.i.. Both radioligands were cleared via the hepatobiliary system, resulting in markedly elevated uptake values in the digestive system, in particular the intestine. However, the intestinal uptake of **[^99m^Tc]2** was three times higher than for **[^99m^Tc]1** (16 ± 7.4 %ID/g for **[^99m^Tc]1** vs. 49 ± 22 %ID/g for **[^99m^Tc]2**, both at 1 h p.i.). At 4 h p.i., both radioligands demonstrated favorable tumor-to-tissue uptake ratios, except for the tumor-to-liver, tumor-to-intestine, and tumor-to-duodenum uptake ratios of **[^99m^Tc]2**. The tumor-to-liver uptake ratio for **[^99m^Tc]2** was lower or equal to 1 at all time points after injection. Together with its more pronounced intestinal uptake, this made **[^99m^Tc]2** unfavorable compared to the in vivo properties of **[^99m^Tc]1**, which showed a more beneficial in vivo behavior, in particular due to the higher tumor uptake. The specificity of tumor uptake was demonstrated for both radioligands through experiments with preinjection of FAUC468 (100 µg per animal). ^99m^Tc-HYNIC (**[^99m^Tc]1**) and ^99m^Tc-tricarbonyl (**[^99m^Tc]2**) showed significantly reduced tumor uptake at 4 h p.i. (*p* < 0.0001 for (**[^99m^Tc]1,** *p* = 0.0069 for (**[^99m^Tc]2**), comparing preinjected animals with the controls (Figure 3), thereby confirming the highly NTSR1-specific tumor uptake of both radioligands in vivo.

**Figure 4:**
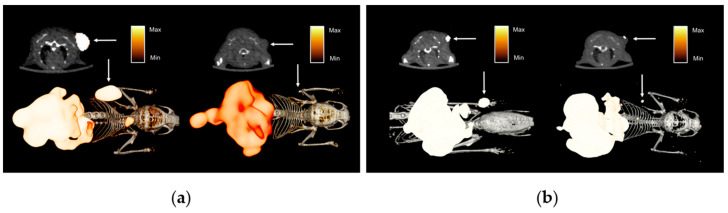
Representative SPECT/CT images (transaxial and coronal view) of HT-29 tumor-bearing nude mice at 4 h p.i., injected with either radioligand (**a**) **[^99m^Tc]1** or (**b**) **[^99m^Tc]2**, respectively, (left side) and coinjected with FAUC468 (100 µg) (right side). Tumors are marked with an arrow. Image reconstruction and analysis were performed using an open-source medical imaging viewer (Horos Version 3.2.1; www.horosproject.org).

The small animal SPECT/CT imaging was performed with both ^99m^Tc-labeled antagonists, using HT-29 tumor-bearing nude mice (Figure 4). The tumor uptake was quantified using a small volume (200 µL) with a defined amount of radioactivity of [^99m^Tc]TcO_4_^−^ (~2 MBq), which was placed near the tail root of the animal. Overall, the distribution of both radioligands determined by SPECT imaging confirmed the results obtained by biodistribution studies. The complex **[^99m^Tc]1** showed significantly higher tumor uptake than **[^99m^Tc]2** at all time points with high tumor retention (Supporting Information Figure S2). Both radioligands reached a maximum tumor uptake at 4 h p.i. (12 ± 2.8 %ID/g (mean ±SD from n = 4) for **[^99m^Tc]1** vs. 3.1 ± 1.1 %ID/g (mean ±SD from n = 4) for **[^99m^Tc]2**) to clearly delineate the tumor on SPECT imaging (Figure 4). Even at 24 h p.i., the tumors were clearly visualized using **[^99m^Tc]1**, whereas the SPECT imaging of animals injected with **[^99m^Tc]2** showed a low signal-to-noise ratio for the tumor at 24 h p.i). Blocking studies once more demonstrated the high specific binding in NTSR1-positive HT-29 tumors of both ^99m^Tc-complexes (Supporting Information Figure S2). Moreover, both radioligands, in particular **[^99m^Tc]2**, displayed pronounced signals in the abdomen, indicating high uptake in the gastrointestinal tract (Figure 4).

## 4. Discussion

^99m^Tc-labeled radiopharmaceuticals are still the most widely used class of radiopharmaceuticals for SPECT diagnostics due to the availability of the approved Mo/Tc generator, the pure gamma decay with an excellent half-life of 6 h, the simplicity of kit synthesis procedures, and their relatively low radiation dose [29,43]. Although PET radiopharmaceuticals are preferred for the primary diagnosis of tumors due to the higher sensitivity of a PET camera compared to SPECT, cost-effective SPECT diagnostics are constantly available in house and could therefore play a valuable role for individual patient pre-selection for radiotherapy and for therapy monitoring, especially in the context of clinical trials.

In the present work, we therefore focused on the preclinical characterization of two ^99m^Tc-labeled NTSR1 ligands in order to complement the previously described therapeutic DOTA-containing ligand ^177^Lu-FAUC468 [28] with a SPECT-suitable ^99m^Tc-ligand as a diagnostic tool, aiming at a theranostic radiopharmaceutical pair. In particular, the possibility of using a ^99m^Tc ligand in SPECT to pre-select patients for subsequent radiotherapy with ^177^Lu-FAUC468 seems to us to be of particular clinical importance.

The xenotransplantion model of NTSR1-positive human HT-29 tumor-bearing nude mice is one of the most frequently used tumor models for the development of NTSR1 radioligands [17,19,23,24,25,26,27,28,44]. Our results demonstrated that in the preclinical SPECT imaging of HT-29 tumor-bearing mice, both the [^99m^Tc]Tc-HYNIC complex **[^99m^Tc]1** and the [^99m^Tc]Tc-tricarbonyl complex **[^99m^Tc]2** showed pronounced nonspecific accumulation in the intestine. This intestinal accumulation was not observed in our previous work with an ^18^F-labeled glycosylated derivative [24] and ^177^Lu-FAUC468 [28].

A comparison with other work in the literature also showed that intestinal accumulation appears to be specific for the linker-chelator moiety of **[^99m^Tc]1** or **[^99m^Tc]2** used, with the more hydrophilic HYNIC complex **[^99m^Tc]1** showing a three times lower intestinal accumulation than **[^99m^Tc]2** at 60 min post-injection. The work of Renard et al. reported NOTA and NODAGA derivatives that showed intestinal accumulation of 4.1–4.3 %ID/g at 2 h p.i. in HT-29 tumor-bearing nude mice [44]. Recently, Lin et al. showed that [^55^Co]Co-NT-Sarcage had a mixed clearance route between the hepatobiliary and renal pathways without substantial intestinal uptake [45]. Other similar studies on metal chelator-containing NTSR1 antagonists of the SR 142948A type, such as tetraazabicyclo[6.6.2]hexadecanediacetic acid (TE2A) derivatives, described increased liver uptake in some cases [46], but substantial intestinal uptake has not yet been reported [47].

It is noteworthy that **[^99m^Tc]1** (log *D*_7.4_ = −0.27) and **[^99m^Tc]2** (log *D*_7.4_ = 1.0) have significantly lower log *D*_7.4_ values than the previously described metal chelator derivatives. This could explain the differences in pKa and solubility, which determines passive permeability and thereby gastrointestinal uptake. This speculation can only be confirmed by in vitro experiments to measure permeability, which we intend to integrate for future studies as an extension of the comparative in vitro assays. It also remains speculative whether the high intestinal uptake observed in the animal model will also be observed in first-in-patient studies and thus might cause diagnostic implications.

Due to its nanomolar NTSR1 affinity, the simple and high-yielding radiosynthesis using the HYNIC precursor **5**, and the excellent tumor uptake in vivo (8.8 ± 3.4 %ID/g at 2 h p.i.), we suggest **[^99m^Tc]1** as a candidate for clinical translation.

^99m^Tc-labeled radiopharmaceuticals are still considered the workhorse of diagnostic nuclear medicine [29]. The radiosynthesis of **[^99m^Tc]1** is based on reaction conditions comparable to those of ^99m^Tc-EDDA/HYNIC-TOC (TEKTROTYD^®^), an approved drug in some EU countries since 2016 [48], and other ^99m^Tc-HYNIC radiopharmaceuticals are being tested in clinical studies [49,50,51,52,53]. Thus, the GMP-compliant synthesis of **[^99m^Tc]1** seems promising for clinical implementation, and GMP validation is currently underway in our laboratory to enable the use of **[^99m^Tc]1** in first-in-patient studies.

In conclusion, **[^99m^Tc]1** has shown advantageous properties compared to **[^99m^Tc]2** at each stage of development, from synthesis to preclinical evaluation. These properties make **[^99m^Tc]1** a valuable candidate for translation into the clinic. Following the successful establishment of a GMP-compliant synthesis and first-in-patient studies, **[^99m^Tc]1** could be used as a highly effective tool for the detection of NTSR1-positive tumors by SPECT imaging.

## Figures and Tables

**Table 1:** Mean values for log *D*_7.4_ and human plasma protein binding (PPB) of **[^99m^Tc]1** and **[^99m^Tc]2**.

Compound	log *D*_7.4_	PPB *	Stability in Human Serum/Human Plasma **
**[^99m^Tc]1**	−0.3	72 ± 1%	73%/94%
**[^99m^Tc]2**	1.0	82 ± 3%	87%/91%

* Data are shown as mean value (%) ± standard deviation (SD) of three independent experiments; ** determined by radio-HPLC after 6 h incubation at 37 °C. The higher tracer stability observed in plasma than in serum seems inexplicable and suggests that a decrease in plasma protein activity may have occurred during storage.

**Table 2:** In vitro binding affinities of compounds **5**, **6 [^nat^Re]2**, and FAUC468 to NTSR1 and NTSR2 expressed as mean K_i_ values (nM) ± SEM (standard error of the mean) from n independent experiments, each determined in triplicate.

Compound	n	K_i_ (NTSR1, nM)	K_i_ (NTSR2, nM)
FAUC468	3	1.9 ± 0.7	100 ± 3
**5**	3	9.5 ± 2.1	270 ± 94
**6**	5	0.32 ± 0.13	85 ± 13
**[^nat^Re]2**	2	2.0 ± 0.3 *	95 ± 6

* K_i_ value in nM ± SD derived from two independent experiments.

## Data Availability

Parts of this study have been reported in the PhD thesis ‘Development and evaluation of neurotensin receptor antagonists labeled with various radionuclides as candidate ligands for PET/SPECT imaging and endoradiotherapy’ by R.P. [54]. All data acquired for this study are stored at the data center of the University Hospital Erlangen, Department of Nuclear Medicine.

## Supplementary Materials

The following supporting information can be downloaded at https://www.mdpi.com/article/10.3390/pharmaceutics17060700/s1, Figure S1: Statistical analysis of the uptake of **[^99m^Tc]1** and **[^99m^Tc]2** in different tissue at different time points obtained from biodistribution data. Figure S2: Statistical analysis of the uptake of **[^99m^Tc]1** and **[^99m^Tc]2** in HT-29 tumors at different time points obtained from SPECT scans.
