# Supplementary material for: 99mTc-Labeled Diarylpyrazoles for Single-Emission Computer Tomography Imaging of Neurotensin Receptor-Positive Tumors: A Comparative Preclinical Study
Source: Pharmaceutics. 2025 May 27;17(6):700. doi: 10.3390/pharmaceutics17060700 (PMC12196348; doi:10.3390/pharmaceutics17060700)
Supplement: Supplementary file 1 [file pharmaceutics-17-00700-s001.zip › pharmaceutics-3607459-supplementary.pdf]

# Supporting information

# $^{99m}$Tc-Labeled Diarylpyrazoles for Single-Emission Computer Tomography Imaging of Neurotensin Receptor-Positive Tumors: A Comparative Preclinical Study

**Roman Potemkin [1,‡], Simone Maschauer [1,6,‡], Harald Hübner [2], Torsten Kuwert [1], Tobias Bäuerle [3,4], Peter Gmeiner [2,5], Olaf Prante [1,5,6*]**

1 Department of Nuclear Medicine, Molecular Imaging and Radiochemistry, Translational Research Center, Friedrich-Alexander-Universität Erlangen-Nürnberg (FAU), 91054 Erlangen, Germany

2 Department Chemistry and Pharmacy, Medicinal Chemistry, Friedrich-Alexander-Universität Erlangen-Nürnberg (FAU), 91058 Erlangen, Germany

3 Department of Radiology, Preclinical Imaging Platform Erlangen (PIPE), Friedrich-Alexander-Universität Erlangen-Nürnberg (FAU), 91054 Erlangen, Germany

4 Department of Radiology, University Medical Center Mainz, Johannes-Gutenberg University, 55131 Mainz; Germany

5 FAU NeW - Research Center New Bioactive Compounds, Friedrich-Alexander-Universität Erlangen-Nürnberg (FAU), 91058 Erlangen, Germany

6 Bavarian Cancer Research Center (BZKF), Translational Research Group TRAFO, 91054 Erlangen, Germany

‡ These authors contributed equally to this work.
* Correspondence: olaf.prante@uk-erlangen.de

| **Content:** | **Page:** |
| --- | --- |
| **Figure S1:** Statistical analysis of uptake of **[$^{99m}$Tc]1** and **[$^{99m}$Tc]2** in different tissue at different time points obtained from biodistribution data | **2** |
| **Figure S2:** Statistical analysis of uptake of **[$^{99m}$Tc]1** and **[$^{99m}$Tc]2** in HT-29 tumors at different time points obtained from SPECT scans | **3** |

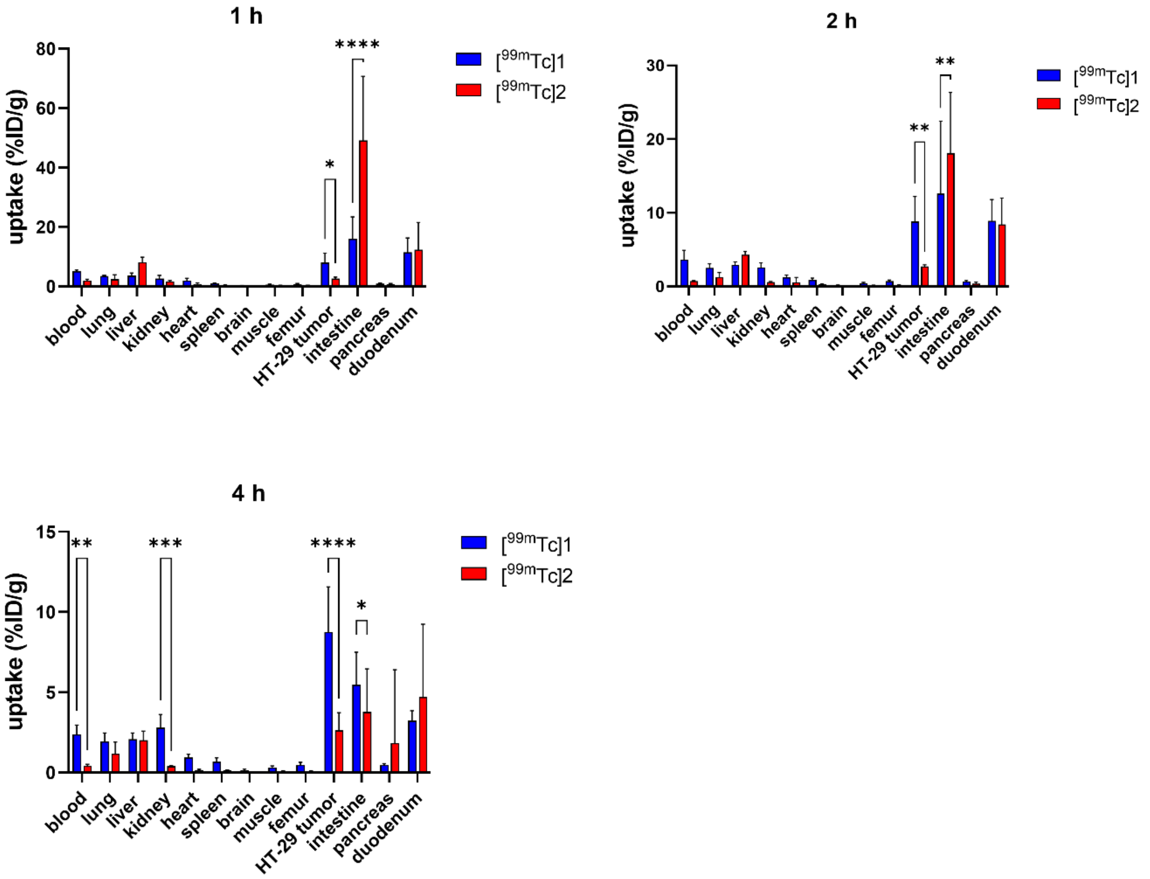


**Figure S1:** Statistical analysis of uptake of **[99mTc]1** and **[99mTc]2** in different tissue at different time points obtained from biodistribution data (mean ± SD, n = 4). Differences in uptake which were statistically significant are indicated by asterisks (Two-Way ANOVA, *$p < 0.05$, **$p < 0.005$, ***$p < 0.0005$, ****$p < 0.0001$).

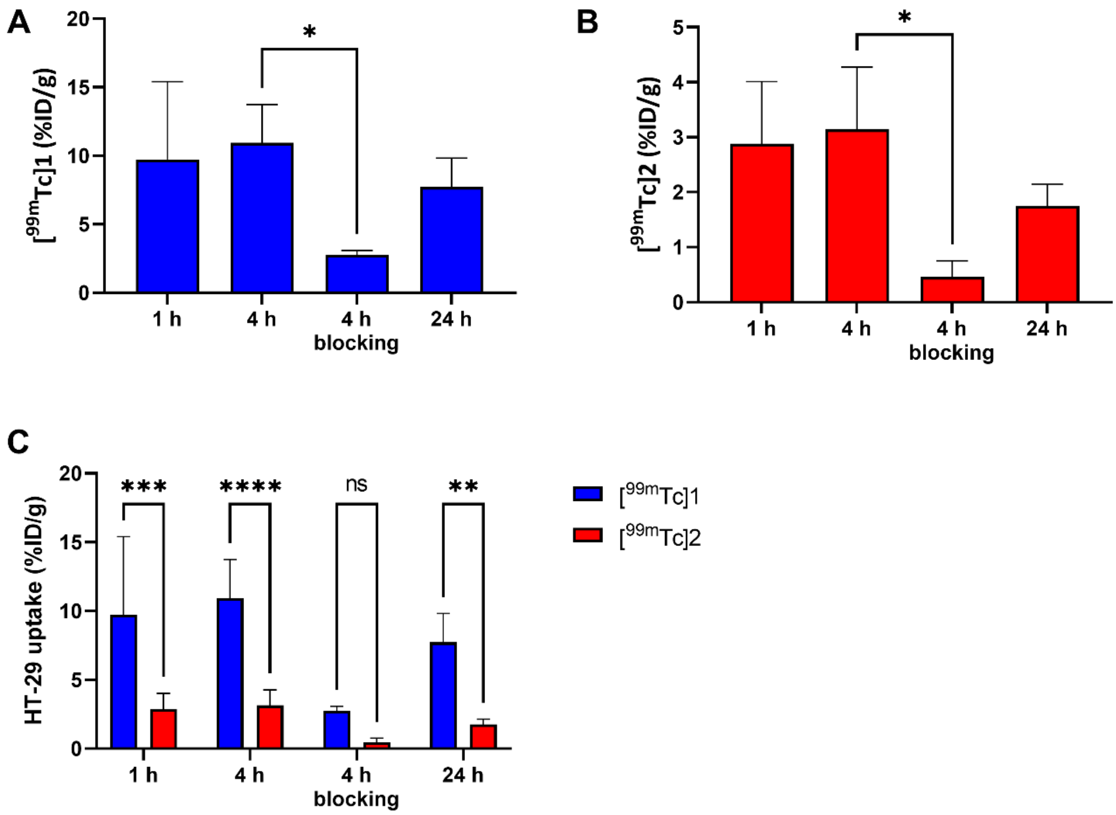


**Figure S2:** Statistical analysis of uptake of **[$^{99m}$Tc]1** and **[$^{99m}$Tc]2** in HT-29 tumors at different time points obtained from SPECT scans (mean ± SD, n = 4). Differences in uptake which were statistically significant are indicated by asterisks (**A**, **B**: paired t-test, **C**: unpaired t-test; *$p < 0.05$, **$p < 0.005$, ***$p < 0.0005$, ****$p < 0.0001$, ns: not significant).
